# Colorimetric enzymatic rapid test for the determination of atropine in baby food using a smartphone

**DOI:** 10.1007/s00216-024-05401-x

**Published:** 2024-07-03

**Authors:** M. Domínguez, D. Moraru, S. Lasso, I. Sanz-Vicente, S. de Marcos, J. Galbán

**Affiliations:** 1 Analytical Chemistry Department, University of Zaragoza, 50009 Saragossa, Spain; 2 Instituto de Nanociencia y Materiales de Aragón (INMA), CSIC-Universidad de Zaragoza, 50009 Saragossa, Spain

**Keywords:** RGB, Tropine, Tropinone reductase, Diaphorase, NADH, Buckwheat

## Abstract

A method for the enzymatic determination of atropine has been developed, which is based on a sequence of reactions involving (1) the hydrolysis of atropine to give tropine; (2) the enzymatic oxidation of tropine with NAD (catalysed by tropinone reductase); and (3) an indicator reaction, in which the NADH previously formed reduces the dye iodonitrotetrazolium chloride (INT) to a reddish species, the reaction catalysed by diaphorase. The method was first developed in solution (linear response range from 2.4 × 10^−6^ M to 1.0 × 10^−4^ M). It was then implemented in cellulose platforms to develop a rapid test where the determination is made by measuring the RGB coordinates of the platforms using a smartphone-based device. The device is based on the integrating sphere concept and contains a light source to avoid external illumination effects. The smartphone is controlled by an app that allows a calibration line to be generated and the atropine concentration to be quantified; moreover, since the app normalizes the CCD response of the smartphone, the results and calibrations obtained with different smartphones are similar and can be shared. Using the G coordinate, the results were shown to have a linear response with the concentration of atropine ranging from 1.2 × 10^−5^ M to 3.0 × 10^−4^ M with an RSD of 1.4% (*n* = 5). The method has been applied to the determination of atropine in baby food and buckwheat samples with good results.

**Graphical Abstract:**

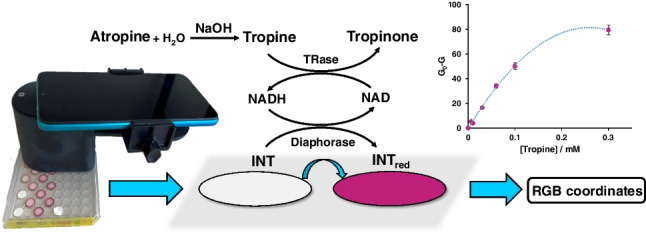

**Supplementary Information:**

The online version contains supplementary material available at 10.1007/s00216-024-05401-x.

## Introduction

Tropane alkaloids (TA) are compounds found in a wide variety of foods and cereals. The most studied of these compounds are atropine (APe) and scopolamine (SPe) [1], due to their applications in the pharmaceutical industry (they act as non-selective inhibitors of muscarinic acetylcholine receptors). Atropine is a pre-anaesthetic, while scopolamine, in the form of butyl-scopolamine, is used to relieve discomfort associated with spasms of the gastrointestinal tract. They are currently extracted from plants, usually genetically modified for their production. However, these compounds can be highly toxic if the established dose is exceeded [1]. Due to adverse health effects, APe and SPe are considered undesirable substances in food and feed (especially for infants), and the European Food Safety Authority (EFSA) has established an acute reference dose of 0.016 mg/kg-day expressed as the sum of APe and SPe [2, 3].

The analytical methods currently used for the determination of TA are based on the use of techniques such as gas chromatography, high-performance liquid chromatography and liquid chromatography coupled to mass spectrometry, capillary electrophoresis techniques and immunoassay techniques [4–6]. The use of liquid chromatography-tandem mass spectrometry has been shown to be a highly sensitive technique for the identification of these metabolites, achieving detection limits below 5 ng/ml [5]. While these techniques provide optimal results, they are slow procedures for quality control so the design and development of rapid methods that require sample treatment is a challenge that needs to be addressed. In this regard, although the development of chemical sensors is increasing in importance, most of those that have been developed so far for the determination of atropine are based on electrochemical [7] or fluorescence quantum dots [8] sensors and lack enough specificity to be applied to real samples.

This lack of specificity could be overcome with the use of immunoassays for AT that give reliable results. However, to develop more accessible and simpler methods for APe that can provide an initial assessment of food toxicity, it would be necessary to focus on enzymatic methods (because of their specificity) and colorimetric methods (because of their accessibility). To the best of our knowledge, no enzymatic methods have yet been proposed for these compounds, probably due to the lack of commercially available suitable enzymes.

**Fig. 1:**
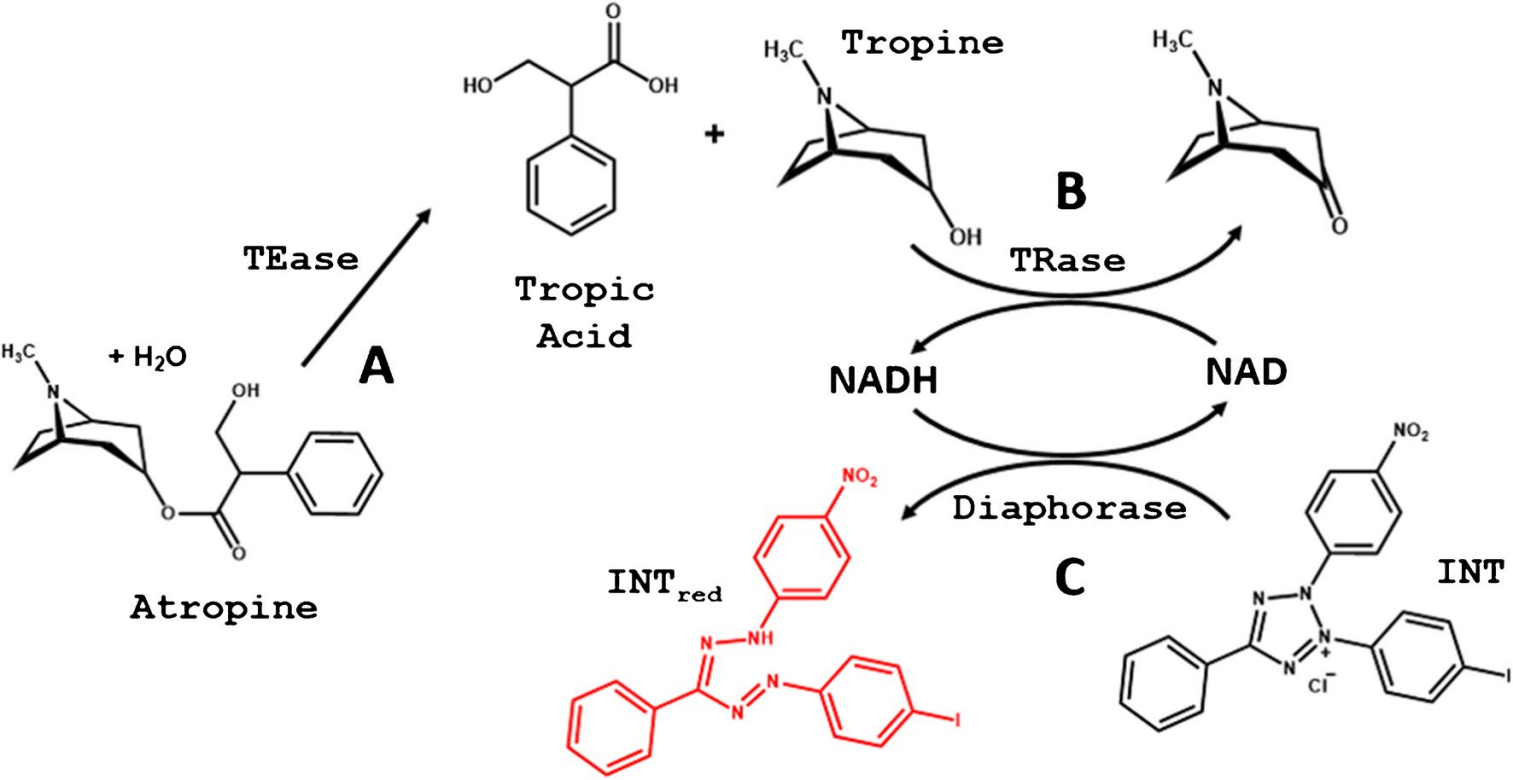
Scheme of the reactions of enzymatic degradation of atropine. **A** Desterification of APe using TEase. **B** Oxidation of tropine using TRase and NAD as a cofactor. **C** Reduction of the dye using the enzyme diaphorase

The possible routes existing for enzymatic reactions involving APe were reviewed. The first step is hydrolysis, which is catalysed by tropine esterase (TEase) (Fig. 1A) [9]. After that, two degradation pathways with highly specific enzymes can then be followed, either via tropic acid or tropine. Via tropic acid, only a complex reaction involving ATP and CoA (catalysed by L-firefly luciferin-CoA ligase) [10] has been found in the literature; this reaction does not allow the coupling of affordable indicator mechanisms. A simpler scheme can be found via tropine; this involves the use of the tropinone reductase (TRase), which catalyses the oxidation of the –OH group using NAD as a cofactor (Fig. 1B). This reaction can be monitored by measuring the molecular absorption/fluorescence properties of NADH (340 nm) [9].

However, better analytical figures of merit can be obtained by submitting the NADH to a further reaction involving the oxidation of tetrazolium salts (such as iodonitrotetrazolium chloride (INT)) to the reddish garnet formazan compound, catalysed by diaphorase (dihydrolipoyl-dehydrogenase). Tetrazolium salts are widely used for both the determination of NADH in cell viability studies [11] and the determination of diaphorase.

The implementation of detectors in low-cost equipment such as cameras, mobile phones, webcams, or digital scanners constitutes a new analytical methodology with characteristics of fast radiation reading and direct signal processing at a moderate cost [12]. Nowadays, this type of analytical platform is experiencing a huge increase, competing against determinations through molecular spectrophotometry, fluorescence and derived sensors, due to the implementation of an analytical methodology based on the use of colour spaces [13]. This implementation is aimed at areas which demand a large amount of analysis and the rapid obtaining of analytical information, such as clinical diagnosis or “in situ” control of food [14].

This paper describes a reliable colorimetric enzymatic method for the determination of APe based on the scheme described in Fig. 1. This method is further implemented on solid supports where the RGB coordinates are acquired by a mobile phone uploaded with a Apk developed in the research group [15]. The Apk includes a correction method that standardizes each measurement making it comparable between different days and phones.

## Materials and methods

### Reagents and solutions

Diaphorase (Sigma-Aldrich D5540-300U), iodonitrotetrazolium chloride, INT (Sigma-Aldrich I8377), tropine (Sigma-Aldrich 93,550), tropinone reductase (TRase) (Gecco Biotech, EC 1.1.1.206), tropine esterase (TEase) (Gecco Biotech, EC 3.1.1.10), β-nicotinamide adenine dinucleotide hydrate NAD (Sigma-Aldrich N1511), atropine sulphate monohydrate (Sigma-Aldrich A0257) and microcrystal cellulose in powder 20 µm (Sigma-Aldrich 310,697) were used. All other reagents were of analytical grade and used without further purification.

### Equipment

A PerkinElmer Lambda 465 UV–vis spectrophotometer (diode-array) and SPECORD 210 Plus UV–vis molecular absorption spectrophotometer were used for UV–vis absorbance measurements. The RGB measurements were performed using with a Xiaomi Mi A2 smartphone with the application ColorGrab™ v. 3.9.2 (Loomatix ©) and the Apk AppColorimetryV1.

### Measurement procedure

#### Method in batch (UV–vis spectrophotometer)

Absorbance measurements were carried out in a UV–vis molecular absorption spectrophotometer. For this purpose, PMMA cuvettes of 1 cm path length were used where the final volume was 2 ml. Measurements were made in spectral scan mode measuring the spectrum from 290 to 690 nm, taking measurements every 10 s.

Twenty microliters of the corresponding atropine solution was mixed with 20 μl of NaOH 2 M in the PMMA cuvettes, and the hydrolysis was allowed to proceed for 5 min. Then, 1615 μL of carbonate buffer pH 10, 40 μl of NAD 5 × 10^−2^ M (in aqueous solution), 5 μl of TRase 8.8 mg/ml (in buffer pH 7.5), 200 μL of dye (INT) 2 × 10^−3^ M (in aqueous solution) and 100 μl of diaphorase 11.2 U/ml were added, and the absorbance at 500 nm was monitored.

#### Method with solid supports

##### Cellulose support synthesis

A 5% (w/V) cellulose suspension was prepared by weighing 50 mg of 20 μm cellulose in a vial, adding the reagents (3.3 × 10^−3^ M NAD, 3.9 × 10^−4^ M INT and 0.67 U.ml^−1^ diaphorase final concentrations) and making up to 1 ml final volume. Seventy-five microliters was added to each well of the well-plate and incubated at 35 °C to dryness (about 2 h).

Use of the RGB coordinates.

The values obtained in the RGB coordinates (*E*_(R,G,B)_) are given by [16]:

1 $${E}_{(R,G,B)}=A\sum_{\lambda }{I}_{\lambda }{P}_{\lambda }{R}_{\lambda }$$

In this equation, *A* is a parameter that includes factors related to the device (camera design, solid angle, light-to-voltage conversion and analogue-to-digital), *I*_λ_ is the spectral power of the light source which is usually known, and *P*_λ_ is the spectral sensitivity of the detector (camera). *R*_λ_ is the reflectance of the solid cellulose support containing the enzymatic reaction; it depends on the analyte concentration according to a second-order mathematical equation derived from the Kubelka–Munk approach. Finally, to avoid both the stray light and the constant A effect in the analytical signal, the following quantitative parameters are finally used, where (*E*_0,(R,G,B)_) is the blank signal [17]:

2 $${\Delta E}_{(\text{R},\text{G},\text{B})}={E}_{0,(\text{R},\text{G},\text{B})}-{E}_{\left(\text{R},\text{G},\text{B}\right)}$$

3 $${\Delta E}_{\left(\text{R},\text{G},\text{B}\right),\text{r}}=\frac{{E}_{0,(\text{R},\text{G},\text{B})}-{E}_{(\text{R},\text{G},\text{B})}}{{E}_{0,(\text{R},\text{G},\text{B})}}$$

##### Colour measurements of the cellulose support

**Fig. 2:**
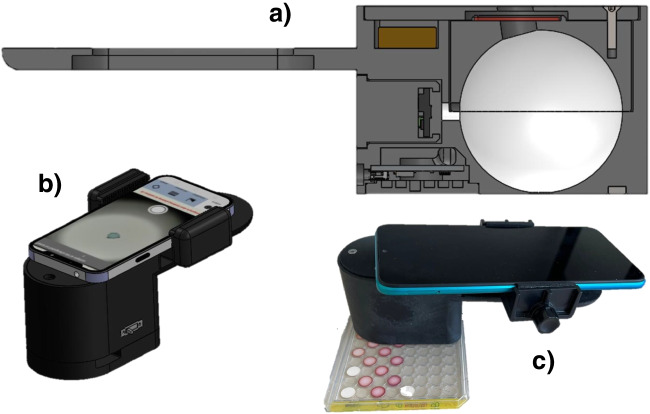
**a** Scheme of the lighting box; **b** lighting box coupled to a smartphone, **b** measures in the solid support with the smartphone-lighting box device

A light box with a universal holder for any mobile phone was developed in our laboratory [18] (Fig. 2). The Xiaomi Mi A2 mobile used in this work was loaded with the application (*AppColorimetryV1*) developed in our laboratory. This application can be downloaded for free and works with the Android operating system (download link: https://drive.google.com/drive/folders/1Hub0fYkR0tabdXlaZeWeYzVLDwll5KzC?usp=drive_link).

The *AppColorimetryV1* features a correction method that standardizes each measurement to a common and well-defined RGB system which significantly reduces measurement errors and variance between different mobile phones, making measurements comparable [13]. The application also allows calibration lines to be performed. The G coordinate is chosen because it provides better results, and G_0_-G (G_0_ and G being the blank and sample signals, respectively) is used as the analytical parameter.

Three replicates of both blank and samples were made in each study. Measurements were taken after 30 min of reaction.

#### Sample treatment: lixiviation from the buckwheat sample

1.5 g of buckwheat is weighed, lixiviated with 30 ml of Milli-Q water in a beaker and left to leach for half an hour. The sample was then centrifuged for 20 min at room temperature at 6000 rpm. The solution was then separated, neutralized to basic pH by the addition of NaOH, and centrifuged again for 20 min (6000 rpm). Finally, the filtered solution was separated, neutralized with HCl, and filtered (by gravity). The filtered solution was washed, and the washing waters were mixed with the solution and bringing to 50 ml in a volumetric flask with Milli-Q water [19].

## Results

### Enzymatic method for the determination of atropine in solution

As shown in Fig. 1, the proposed method consists of three steps: de-esterification of atropine to tropine, oxidation of tropine catalysed by TRase and a colorimetric reaction.

The de-esterification step was first studied using TEase. This enzyme (as well as TRase) was produced specifically for our laboratory by Gecco Biotech B.V., and although the formation of tropine was observed, the transformation was not quantitative enough even though several experimental conditions were tested. The best results were obtained when the hydrolysis was carried out at basic pH (without the use of the enzyme) with NaOH; ESM 1 shows the results of the hydrolysis optimization.

The oxidation reaction of tropine by TRase was then studied by measuring the absorbance at 340 nm (NADH). The experimental results obtained during the optimization study of this reaction, involving TRase concentration, pH and temperature, are detailed in the supplementary material section (ESM2). Finally, the colorimetric reactions involving the oxidation of NADH by INT and the formation of the reddish INT_red_ were studied. ESM3 summarizes the results obtained during the optimization study, which included diaphorase and INT concentrations and pH. Under the optimal conditions found (see the “Method in batch (UV–vis spectrophotometer” section), the analytical figures of merit obtained are summarized in Table 1 (Fig. S3d shows the UV–vis spectra of INT with increasing concentrations of APe and inside the colour of these solutions). Taking into account that the molar absorptivity of the reduced form of INT is 19.3 mM^−1^ cm^−1^ [20], the enzymatic method gives an atropine conversion of 58%.

**Table 1:** Analytical figures of merit of the method for APe in solution

Sensitivity	Limit of detection (LOD, mol/l)	Limit of quantification (LOQ, mol/l)	Lineal response range (mol/l)	RSD (*n* = 5) [APe] = 1·10^−4^ mol/l)
11.3 mM^−1^ cm^−1^	7.1·10^−7^	2.4·10^−6^	2.4·10^−6^–1·0^−4^	3.8%

The method has been applied to the determination of ATe in baby food. These samples were provided by the Laboratorio de Salud Publica (Government of Aragón-Spain). They had previously analysed the extract (according to UNE EN: 15,662:2019) using a validated HPLC–MS/MS method [21], and the atropine concentrations found were below their detection limit. We then spiked these samples with known concentrations of ATe. The results of the recovery study are shown in Table 2, which validate the ability of this method to analyse real samples.

**Table 2:** Results of the measurements obtained for each APe concentration in solution (all samples were analysed in triplicate)

[APe] added mol/l	% Recovery (Method in solution)	% Recovery (Cellulose test method)
1·10^−5^	111 (± 6) %	–-
6·10^−5^	93 (± 2) %	88 (± 3) %
1·10^−4^	97 (± 2) %	108 (± 4) %

### Cellulose test platforms

#### Measurement system: G coordinate

The ultimate aim of this study is to provide a quick and easy test for the determination of atropine in food. With that objective, this methodology has been implemented in cellulose test platforms, where the concentration of APe could be determined by measuring the RGB coordinates using a smartphone-based device system developed in our laboratory. The main improvements of this system compared to others previously proposed are as follows:

- The light box is based on the integrating sphere concept, which allows more light to be collected and is more reproducible, so that lower RGB values can be taken with better precision.
- It is fixed to the smartphone with a universal support (compatible with most of the smartphones on the market).
- The colour measurement is carried out by means of an application (AppColorimetryV1) developed in our laboratory which can be freely loaded on the mobile. This app allows (1) correcting the differences between mobiles by means of a correction matrix (calculated specifically for each mobile) that normalizes the RGB values obtained and makes them comparable between mobiles, (2) performing second-order calibration lines from the RGB values measured for the standards and (3) determining the concentration of atropine from the image taken from the samples.

The maximum absorbance of INT_red_ occurs at 500 nm. This wavelength corresponds to the green light of the visible spectra, so it is expected that the G coordinate will give the best sensitivity. The experimental results agree with this hypothesis (ESM4, Table S4a), and this coordinate gave about 3 times higher sensitivity than B or R.

#### Optimization

Cellulose was used as a solid substrate for the preparation of the platforms. A suspension containing the appropriate concentration of cellulose and reagents (see 2.2.3) was prepared. Previous studies carried out by our research group with this material have shown that the best results are obtained with dispersions containing 3% (w/w) or 5% (w/w) cellulose [22]; a lower % gives weak supports and higher concentrations do not allow reproducible preparation. In this reaction, both cellulose concentrations gave similar sensitivity (G_0_-G values of 133 and 138 for 3% and 5%, respectively), but the uncertainty expressed as RSD was worse for 3% cellulose (RSD = 9%), compared with RSD = 0.5% for 5% cellulose (*n* = 5 in both cases).

Different concentrations of INT, diaphorase and NAD were tested by addition to the 5% cellulose suspensions. These concentrations were tested at two levels of atropine concentration in order to make an initial assessment of the sensitivity they could give. The results obtained are shown in ESM4 (Tables S4b and S4d) which allow the optimal concentrations to be derived.

Although it was possible to immobilize TRase simultaneously with the rest of the reagents, it was better to add TRase to the previously synthesized cellulose platforms. There are two main reasons for this: (1) some activity of this enzyme is lost during entrapment, and (2) during drying in the presence of the enzyme, the supports acquire a very high surface tension, and this prevents a homogenous impregnation of the cellulose by the analyte.

#### Cellulose test stability

The stability of the sensor was checked over time. To do this, several groups of three sensors (for testing 0 M, 3.3 × 10^−5^ M and 3.3 × 10^−4^ M atropine concentrations) were prepared and subjected to the reaction during 4 consecutive days; no significant differences were observed between these measurements (Table S4f). These results show that the cellulose supports were preserved for at least 5 days after their preparation.

#### Effect of ionic strengths

The effect of ionic strength was studied by adding different concentrations of NaCl during the preparation of the cellulose suspensions. As done in the “Optimization” section, each concentration of NaCl was tested for two levels of atropine concentration to check how it affects the sensitivity. The results obtained are shown in ESM4 (Table S4g). These results show that the ionic strength does not cause any changes in the sensor signal for concentrations within the linear range, while a loss of signal is observed at higher concentrations.

#### Analytical figures of merit

**Fig. 3:**
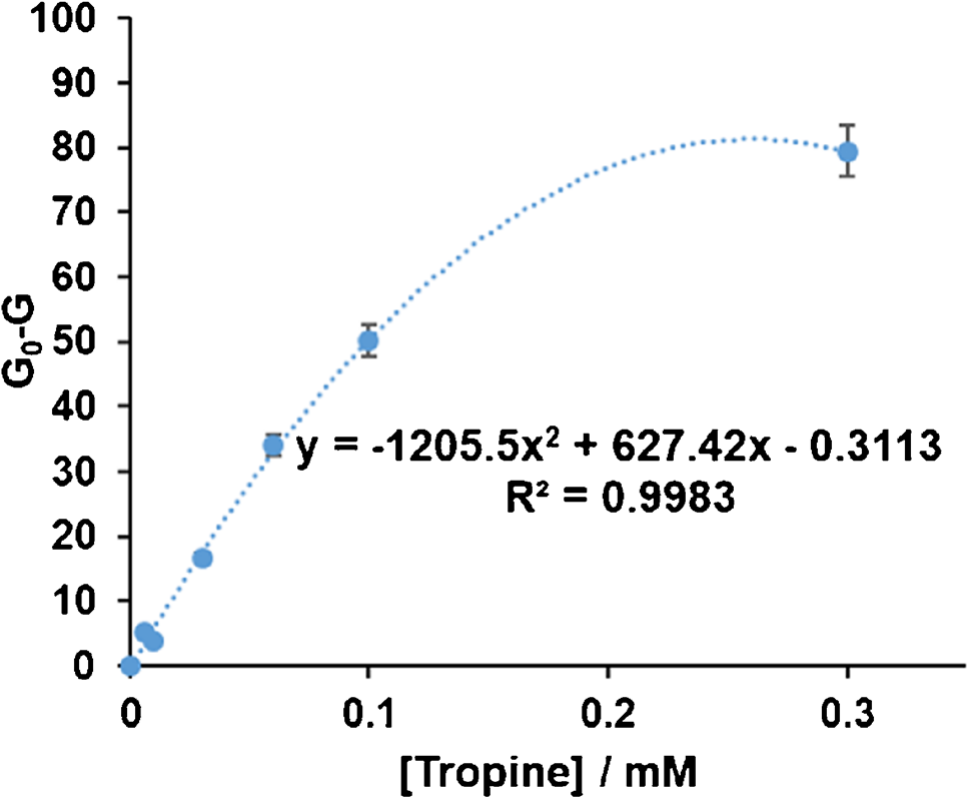
Calibration curve obtained for different tropine concentrations. Experimental conditions as indicated in 2.3.2.1

**Fig. 4:**
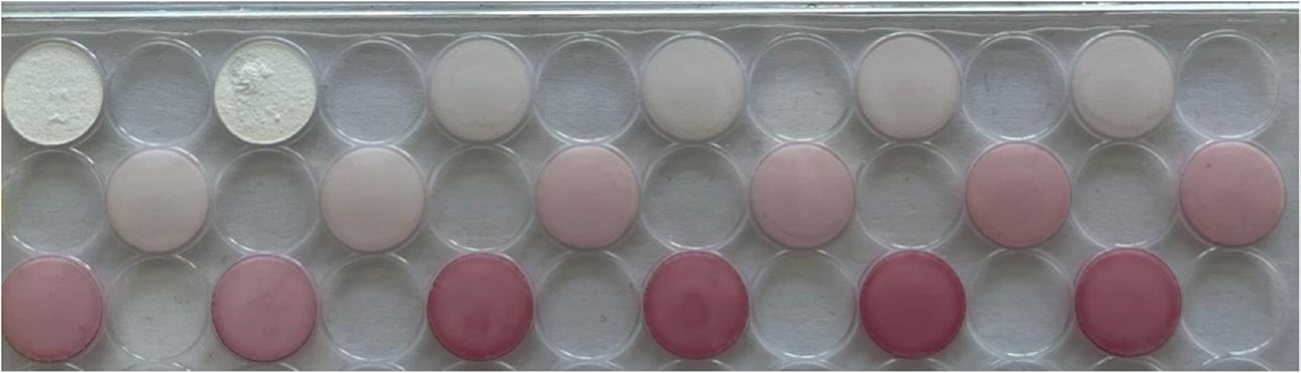
Colour obtained for the different APe concentrations used during the calibration curve. Each concentration in duplicate. Experimental conditions as indicated in 2.3.2.2

Figure 3 (and ESM 5, Table S5a) shows the results obtained during the calibration study; each measurement is the average of three different values. From these values, the LOD and LOQ were obtained. An example of the colours obtained is shown in Fig. 4. Table 3 compiles the complete analytical figures of merit of this method.

**Table 3:** Analytical figures of merit of the method in cellulose platforms

Linear range	LOD	LOQ	RSD (*n* = 3)	
(mol/l)	(mol/l)	(mol/l)	[APe] = 3·10^−5^ mol/l	[APe] = 3·10^−4^ mol/l
1.2·10^−5^–3·10^−4^	4.1·10^−6^	1.2·10^−5^	2.6%	0.7%

#### Application of the method to real samples

The method was first applied to the determination of APe in the spiked samples previously indicated (Table 2) demonstrating the ability of these platforms to determine atropine in real samples.

The method was then applied to a sample of buckwheat that had previously been subjected to extraction as described in the “Sample treatment: lixiviation from the buckwheat sample” section. The cellulose test platforms were prepared as described above, and, after drying, the buckwheat extract was submitted to the standard addition method; this determination was carried out in triplicate. The result obtained was a concentration in buckwheat of 0.0612 ± 0.0016 g/kg, in agreement with normal values reported by the EFSA (2).

## Conclusions

A novel rapid test for the enzymatic determination of APe has been successfully developed. It has been demonstrated, for the first time, that APe can be determined by coupling the enzymatic reaction with TRase to an indicator reaction. This methodology can be successfully implemented in cellulose platforms to determine APe concentrations in foods using the G coordinate proving to be a rapid and reliable method.

## Supplementary Information

Below is the link to the electronic supplementary material.

Supplementary file1 (DOCX 586 KB)
