# Supplementary material for: Colorimetric enzymatic rapid test for the determination of atropine in baby food using a smartphone
Source: Anal Bioanal Chem. 2024 Jul 3;416(30):7317–23. doi: 10.1007/s00216-024-05401-x (PMC11584467; doi:10.1007/s00216-024-05401-x)
Supplement: Supplementary file 1 — Supplementary file1 (DOCX 586 KB) [file 216_2024_5401_MOESM1_ESM.docx]

**Electronic Supplementary Material:**

**Colorimetric enzymatic rapid test for the determination of Atropine in baby food using a Smartphone**

M. Domínguez, D. Moraru, S. Lasso, I. Sanz-Vicente, S. de Marcos, J. Galbán

Analytical Chemistry Department, University of Zaragoza, Zaragoza 50009, Spain.

Instituto de Nanociencia y Materiales de Aragón (INMA), CSIC-Universidad de Zaragoza, Zaragoza 50009, Spain

**ESM1.- Deesterification of APe**

20 μL of APe were mixed with 20 μL of NaOH 2 M for different times. 40 μL of the resulting solution were diluted in 2 mL of 0.1M (pH=7) phosphate buffer containing the other reactants required for the reaction (ESM2). As observed in Figure S1, for hydrolysis times above 4 minutes, the signal obtained in the subsequent reaction (tropine oxidation) showed no variation. Therefore, a hydrolysis time of 5 minutes is chosen as optimal to ensure that this reaction has been completed.


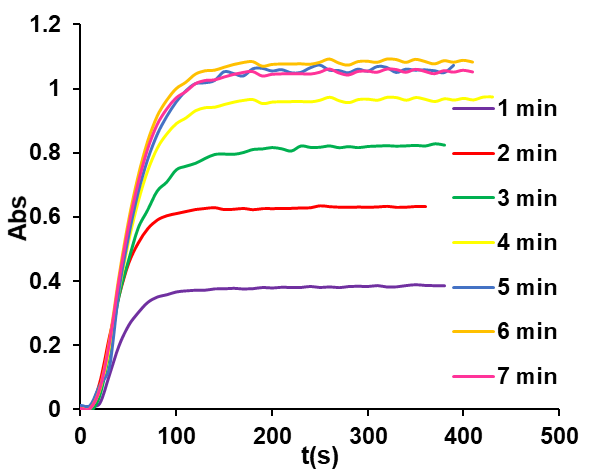


**Figure S1:** Variation of the Abs(340 nm) nm with the reaction time for different hydrolysis times.

**ESM2.- Results obtained during the optimization of the NADH method.**

The effect of the TRase concentration, the pH and the temperature on the enzymatic oxidation of tropine was studied. The absorbance was always measured at 340 nm. The NAD concentration used for all the assays is 10^-3^ M. This concentration is chosen to be in high excess with respect to the tropine concentration. Using a tropine concentration of 10^-4^ M, the results of the optimizations are shown in Figures S2a, S2b and S2c. Table S1 compiles the optimal conditions found.


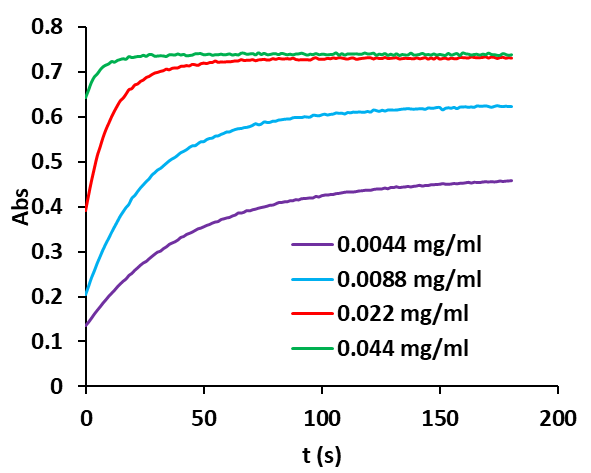


**Figure S2a:** Effect of the TRase concentration


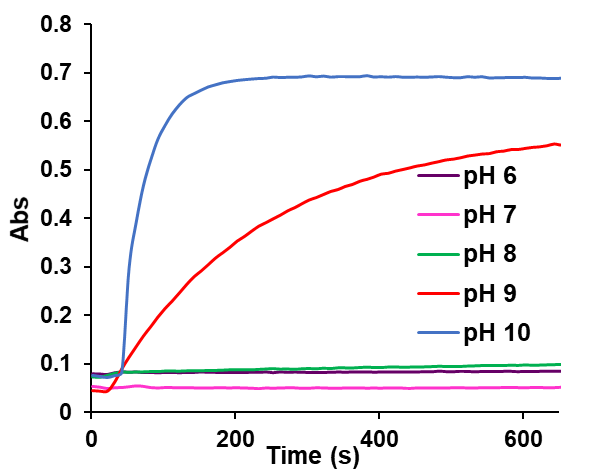


**Figure S2b:** Effect of the pH.


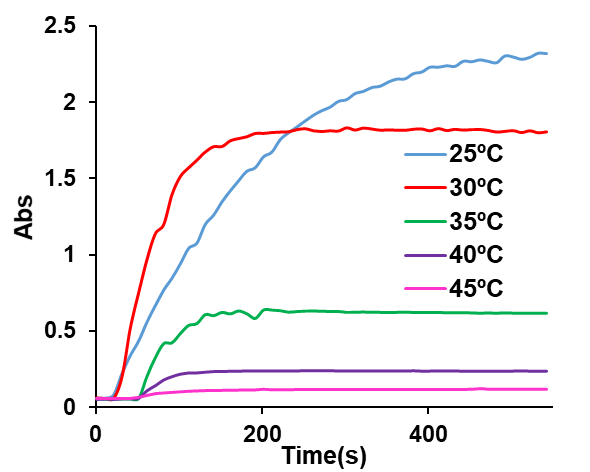


**Figure S2c:** Effect of the temperature of the reaction. In that assay the tropine concentration was 10^-4^ M.

**Table S1:** Optimized conditions for the reaction of oxidation of tropine.

| pH | Temperature | [NAD] | [Tropinone Reductase] |
| --- | --- | --- | --- |
| 10 (Carbonate buffer 0,1 M) | 25ºC | 1·10^-3^ M | 2.2·10^-2^ M |

**ESM3.- Optimization of the indicator reaction**

To optimize the reaction between the NADH (produced by the degradation of the tropine) and INT , the absorbance is measured at 500 nm (maximum absorption of the dye) and [NADH]=10-4M.

**1) pH optimization**. Studies were carried out with the following buffer solutions: 0.1 M carbonate (pH 5, 9 and 10) and 0.1 M phosphate (pH 6, 7 and 8).


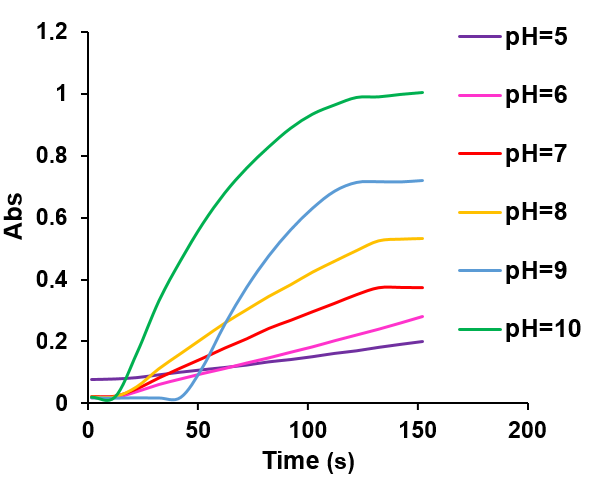


**Figure S3a:** Variation of the absorbance at 500 nm with the reaction time at different pHs [Diaphorase=0.56 U/mL], [INT]= 1*10^-4^ M and T= 25ºC.

pH 10 with carbonate buffer solution 0.1 M is chosen as the optimal pH, which also coincides with the optimal pH of the reaction to which it is to be coupled.

**2) Temperature**. Tested between 25 and 50ºC at 5ºC intervals. No difference was found. The optimum temperature was 25ºC (the same as the previous reaction).

**3) Diaphorase**.


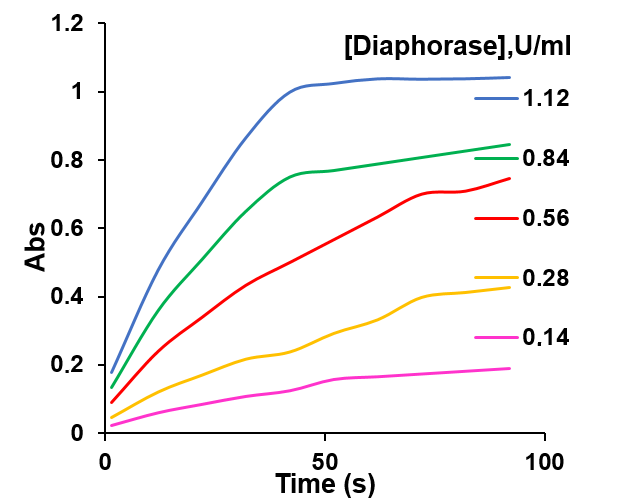


**Figure S3b:** Variation of the absorbance at 500 nm with the reaction time for different concentrations of diaphorase. [pH=10 Carbonate, [INT]= 1*10^-4^M and T= 25ºC.

**4) INT**


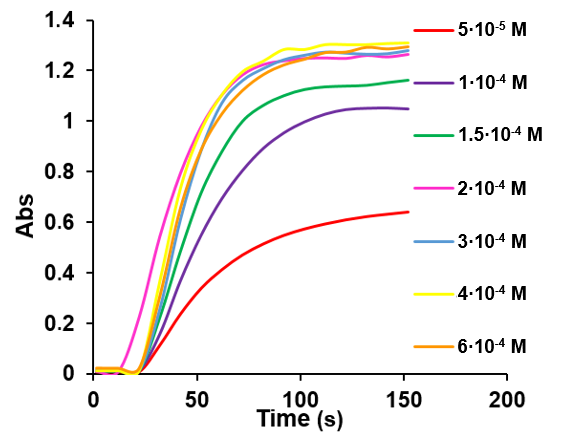


**Figure S3c:** Variation of the absorbance at 500 nm with the reaction time for different concentrations of dye. pH=10 Carbonate, [Diaphorase]= 0,56 U/ml and T= 25ºC].

2·10^-4^ M was chosen as optimal.

**5) Calibration line for Ape determination.**

With the conditions optimized conditions, both reactions are coupled to check that they are working correctly. The aim is that the NADH produced as a product in the first reaction acts as a reactant in the second.

The reaction worked properly so the optimization of the concentration of the two enzymes was repeated in the same way as in the previous section to verify that the optimal conditions did not vary when coupling both reactions.

Once it had been verified, a calibration curve was constructed by making measurements for different atropine concentrations keeping the rest of the optimized variables constant (Tª=25ºC, pH=10 carbonate, [NAD]=10^-3^ M, [TRase]=0.022mg/ml, [INT]=2·10^-4^ M, [Diaphorase]=0.56U/ml). Absorption measurements were taken at 500 nm. The variation of the INT spectra with the concentration of atropine and the calibration curve can be seen in Figure S3d and 3e.

**Figure S3d**: INT spectra obtained with increasing concentration of APE and the colour of these solutions


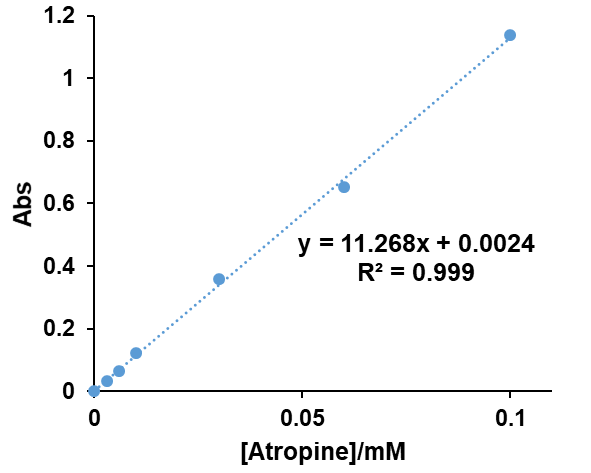


**Figure S3e:** Calibration curve for atropine taking absorption measurements at 500 nm.

**ESM4.- Optimization of the cellulose platforms**

**A) Studying the RGB coordinate**

Table S4a shows the RGB values obtained using 5% cellulose supports; the G coordinate shows the highest sensitivity. In addition, G0-G shows the highest reproducibility. Therefore, G_0_-G was chosen as the parameter to work with solid supports.

**Table S4a:** RGB coordinates obtained for 5% cellulose supports.

| Coordinate | Value | s | RSD (%) |
| --- | --- | --- | --- |
| R | 151 | 7.07 | 4.68 |
| G | 134.5 | 0.70 | 0.53 |
| B | 136.5 | 4.95 | 3.63 |
| R_0_-R | 48 | 7.07 | 14.73 |
| G_0_-G | 138.5 | 0.70 | 0.51 |
| B_0_-B | 51.5 | 4.95 | 9.61 |

**B) Optimization of the INT concentration**

**Table S4b:** G_0_-G coordinate measured for each dye concentration (n=5)

| [INT]  (mol/l) | Tropine (mol/l) | |
| --- | --- | --- |
|  | 3.33·10^-5^ | 3.33·10^-4^ |
| 3.95·10^-4^ | 23±12 | 133.3±5.8 |
| 7.91·10^-4^ | 26.7±8.5 | 129.3±5.8 |
| 1.18·10^-3^ | 32±10 | 134.7±1.1 |
| 1.78·10^-3^ | 38.3±2.1 | 139.3±9.9 |
| 2.37·10^-3^ | 43.0±7.6 | 142.7±14.2 |

Representing the G_0_-G parameter as a function of tropine concentration for the different dye concentrations gives the slopes shown in Table S4c. It can be observed that the concentration of INT that presents a greater sensitivity is that of 3.95·10^-4^ M, therefore, this will be the concentration used in this work.

**Table S4c:** Slope obtained for each dye concentration.

| **[INT] (mol/l)** | **Slope (l/mol)** |
| --- | --- |
| 3.95·10^-4^ | 368146 |
| 7.91·10^-4^ | 342565 |
| 1.18·10^-3^ | 343677 |
| 1.78·10^-3^ | 337004 |
| 2.37·10^-3^ | 332555 |

**C) Optimization of the Diaphorase concentration**

**Table S4d:** G_0_-G coordinate measured for each diaphorase concentration (n=5)

| [Diaphorase] (U/mL) | Tropine (mol/l) | |
| --- | --- | --- |
|  | 3.33·10^-5^ | 3.33·10^-4^ |
| 0 | 33.3±5.7 | 120.7±3.0 |
| 0.67 | 33.7±5.9 | 156±13 |
| 1.12 | 40.3±4.2 | 152±10 |
| 1.57 | 44.6±3.9 | 160±12 |
| 2.02 | 32.3±9.7 | 159.3±3.8 |

Representing the G_0_-G parameter as a function of tropine concentration for the different diaphorase concentrations gives the slopes shown in Table S4e. It can be observed that the concentration of diaphorase concentration that presents a greater sensitivity is that of 2.02 U/ml.

**Table S4e:** Slope obtained for each diaphorase concentration.

| [Diaphorase] (U/ml) | Slope (ml/U) |
| --- | --- |
| 0 | 291403 |
| 0.67 | 408186 |
| 1.12 | 372595 |
| 1.57 | 387054 |
| 2.02 | 423757 |

**D) Optimization of the NAD concentration**

Finally, NAD was also immobilized, so that the solution added to the celluloses contains only tropine and buffer solution. The results show that the reaction has a greater sensibility using a NAD concentration of 3.33·10^-3^ M.

**Table S4f:** G_0_-G coordinate for each day during the experiment.

| [Tropine], mol/l | Day 1 | Day 2 | Day 3 | Day 4 | Day 8 | Day 11 | Day 15 | Day 20 |
| --- | --- | --- | --- | --- | --- | --- | --- | --- |
| 0 | 0 | 0 | 0 | 0 | 0 | 0 | 0 | 0 |
| 3.33·10^-5^ | 16.22 | 24.36 | 20.52 | 22.08 | 23.13 | 20.17 | 22.12 | 18.23 |
| 3.33·10^-4^ | 76.54 | 73.41 | 65.82 | 74.94 | 72.56 | 64.28 | 73.34 | 62.41 |

**Table S4g**: Effect of ionic strength for different tropine concentrations.

| [NaCl] (mol/l) | Tropine (mol/l) | |
| --- | --- | --- |
|  | 3.33·10^-5^ | 3.33·10^-4^ |
| 0 | 29.1±3.9 | 95.1±2.7 |
| 0.05 | 35.2±1.9 | 86.5±2.2 |
| 0.1 | 31.6±3.2 | 80.7±1.4 |
| 0.2 | 34.5±3.5 | 73.1±3.8 |
| 0.3 | 27.1±4.2 | 67.4±2.1 |

**ESM5.- Analytical figures of merit of cellulose platforms.**

**Table S5a:** Results obtained for each tropine concentration in the calibration study. Experimental conditions as indicated in 2.3.2.1

| **[Tropine] (M)** | **G_0_-G** | **s** | **RSD (%)** |
| --- | --- | --- | --- |
| 0 | 0 | 1.32 | - |
| 6·10^-6^ | 5.18 | 0.98 | 0.70 |
| 1·10^-5^ | 3.88 | 1.32 | 0.92 |
| 3·10^-5^ | 16.53 | 1.68 | 1.26 |
| 6·10^-5^ | 34.05 | 2.84 | 2.40 |
| 1·10^-4^ | 50.11 | 1.92 | 1.81 |
| 3·10^-4^ | 79.42 | 2.09 | 2.61 |
